# The Influence of Oxygen Plasma on Methylammonium Lead Iodide (MAPbI_3_) Film Doped with Lead Cesium Triiodide (CsPbI_3_)

**DOI:** 10.3390/molecules26175133

**Published:** 2021-08-24

**Authors:** Shui-Yang Lien, Chi-Wei Wang, Wen-Ray Chen, Chuan-Hsi Liu, Chih-Chieh Kang, Chien-Jung Huang

**Affiliations:** 1 School of Opto-Electronic and Communication Engineering, Xiamen University of Technology, Xiamen 361024, China; sylien@xmut.edu.cn; 2 Department of Materials Science and Engineering, Da-Yeh University, Dacun, Changhua 51591, Taiwan; 3 Fujian Key Laboratory of Optoelectronic Technology and Devices, Xiamen University of Technology, Xiamen 361024, China; 4 Department of Applied Physics, National University of Kaohsiung, Kaohsiung University Rd., Kaohsiung 81148, Taiwan; m1074306@mail.nuk.edu.tw; 5 Department of Electronic Engineering, National Formosa University, Wenhua Rd., Yunlin County 632301, Taiwan; chenwr@nfu.edu.tw; 6 Department of Mechatronic Engineering, National Taiwan Normal University, Heping East Rd., Taipei 10610, Taiwan; liuch@ntnu.edu.tw; 7 Department of Electro-Optical Engineering, Southern Taiwan University of Technology, Nan-Tai Street, Tainan 71105, Taiwan; kangc@stust.edu.tw

**Keywords:** composite perovskite, doped, quantum dots, methylammonium lead iodide, oxygen plasma

## Abstract

In recent years, the study of organic–inorganic halide perovskite as an optoelectronics material has been a significant line of research, and the power conversion efficiency of solar cells based on these materials has reached 25.5%. However, defects on the surface of the film are still a problem to be solved, and oxygen plasma is one of the ways to passivate surface defects. In order to avoid destroying the methylammonium lead iodide (MAPbI_3_), the influence of plasma powers on film was investigated and the cesium triiodide (CsPbI_3_) quantum dots (QDs) were doped into the film. In addition, it was found that oxygen plasma can enhance the mobility and carrier concentration of the MAPbI_3_ film.

## 1. Introduction

Hybrid halide perovskite (HHP) has become a promising candidate for third-generation solar cell absorption layers. Since Kojima et al. [1] first reported it in 2009, this material has shown great potential due to its excellent photovoltaic response. In the past few years, the power conversion efficiency (PCE) of solar cells based on this material has increased from 3.8 percent in 2009 [1] to 25.5 percent [2,3]. The high efficiency of lead-based perovskite solar cells can be attributed to band gaps close to the Shkreli–Quessel limit, high light absorption coefficients, excellent electric mobility and large electric diffusion lengths [4,5]. The performance of HHP solar cells is expected to be greatly improved due to their excellent optical properties. However, polycrystalline perovskite materials show a shorter photoluminescence life, indicating the existence of electric defects [6]. These defects originate from the crystal boundary inside the perovskite, which may capture free-charged carriers. In this article, references are presented that mention that trace amounts of oxygen are beneficial to methylammonium lead iodide (MAPbI_3_) films [7,8]. Moreover, defects can be decreased in number by introducing oxygen. Additionally, oxygen plasma treatment was used to remove excess surface ligand and precursor, and the absorption and electrical performance was also improved. However, excess oxygen ions cause oxidation and ion bombardment [9], leading to a decrease in the stability and structural destruction of the film. Thus, cesium triiodide (CsPbI_3_) quantum dots (QDs) were doped into the MAPbI_3_ film to achieve anion exchange in order to make the overall structure more stable [10], and the absorption was enhanced due to the unique optical properties of the QDs. In addition, the power of the oxygen plasma was tuned to investigate its influence on the MAPbI_3_. For the investigation, X-ray photoelectron spectroscopy (XPS) was used to analyze the surface composition of the MAPbI_3_-doped CsPbI_3_ QDs. Furthermore, it was found that the oxygen plasma was able to improve the carrier concentration and mobility of the MAPbI_3_. This innovative film as a potential material was optimized by oxygen plasma treatment at different powers. In this article, this work provides in-depth details to illustrate the investigation of the formation mechanism and surface properties.

## 2. Results

**Figure 1:**
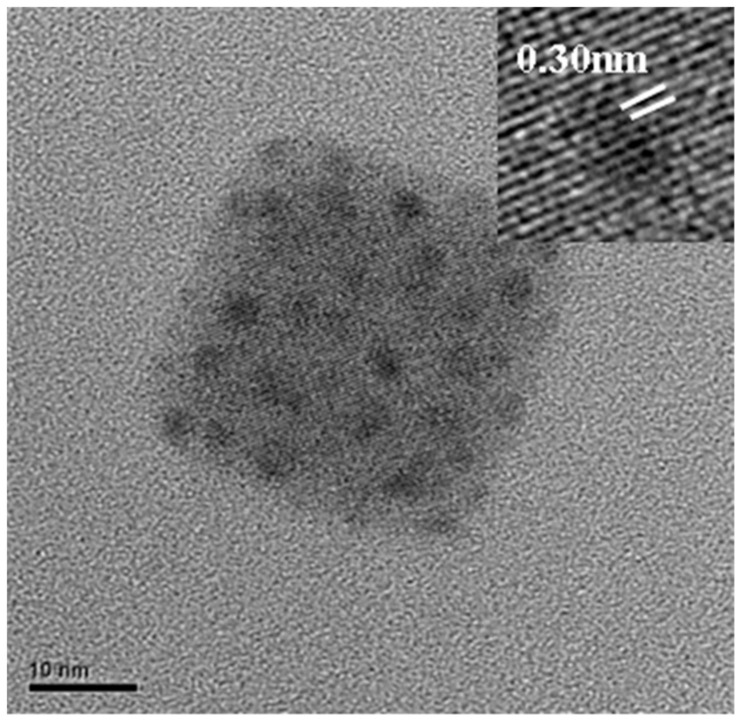
TEM image of the CsPbI_3_ QD.

**Figure 2:**
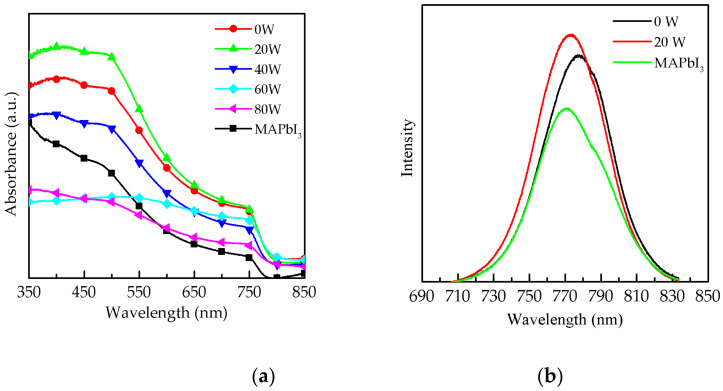
Absorbance spectrum of (**a**) the pure MAPbI_3_ film and the MAPbI_3_ film with CsPbI_3_ QDs treated with oxygen plasma at 0 to 80 W. (**b**) Photoluminescence results of the MAPbI_3_ film and composite perovskite films without and with oxygen plasma treatment at 20 W.

Figure 1 shows the transmission electron microscope (TEM) image of CsPbI_3_ QDs, and the d-space of CsPbI_3_ QDs is 0.30 nm. Figure 2a plots the absorbance spectra of a MAPbI_3_ film without and with CsPbI_3_ QDs and further optimization by means of oxygen plasma treatment at 0 to 80 W. The results show the phenomenon that the absorption of the MAPbI_3_ film was enhanced from 350 to 750 nm after doping CsPbI_3_ QDs, and this is a great improvement in absorption compared to the MAPbI_3_ film without CsPbI_3_ QDs. The reason for this is the excellent optical properties and the wide energy gap of the CsPbI_3_ QDs [11,12]. Multiple studies demonstrated that the light-harvesting ability of the perovskite film could be enhanced by doping with QDs [13,14]. In addition, the surface of the perovskite films was treated with the oxygen plasma at 20 to 80 W. It can be observed that the strongest absorbance of the films was at 20 W, because the excess ligands and impurities were removed from the surface. However, the absorbance gradually decreased in the power range of 40 to 80 W due to the degradation of MAPbI_3_ caused by high-power oxygen ion bombardment [15]. Figure 2b shows the photoluminescence (PL) results of the pure MAPbI_3_ film and MAPbI_3_ films doped with CsPbI_3_ QDs with and without oxygen plasma treatment at 20 W. The PL intensity of the MAPbI_3_ film doped with CsPbI_3_ QDs was drastically enhanced compared to that of pure MAPbI_3_ film due to the Cs ion exchange process [16]. The PL intensity of MAPbI_3_ film with QDs treated with oxygen plasma was also enhanced compared to the MAPbI_3_ film not treated with QDs. This is attributed to the removal of the ligands and impurities. In addition, the reason for the enhancement might be the passivation of surface defects with plasma treatment, and this was caused by the replacement of the defects [17]. It is observed that the luminescence peak of the composite perovskite film exhibits a shift towards higher wavelengths from 770.4 to 776.7 nm, caused by the doping with CsPbI_3_ QDs. In addition, the sample showed a shift to lower wavelengths from 776.7 to 773.2 nm after treatment with oxygen plasma at 20 W, which may be due to the removal of excess ligands and precursors or the decrease in the number of defects on the surface [18,19]. The decrease in the number of defects resulted in the increase in the band gap, leading to the shift in the PL peak.

**Figure 3:**
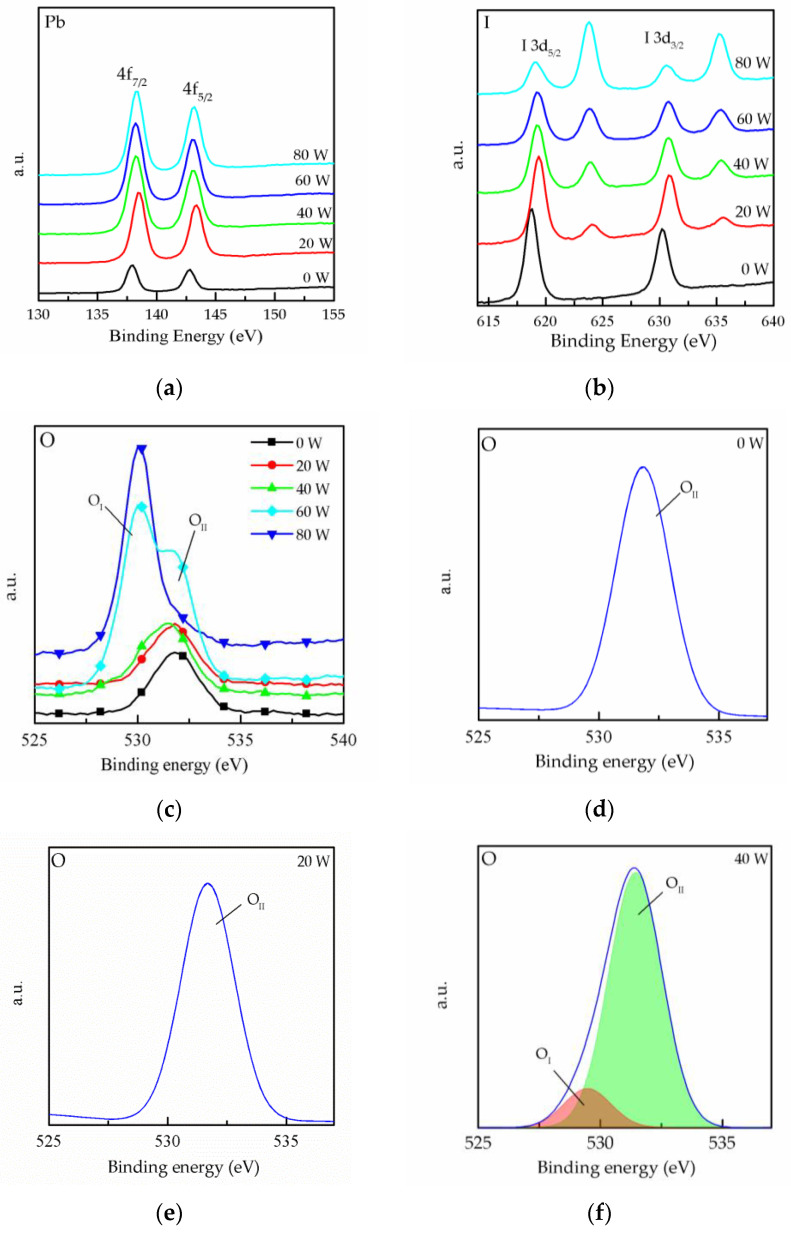

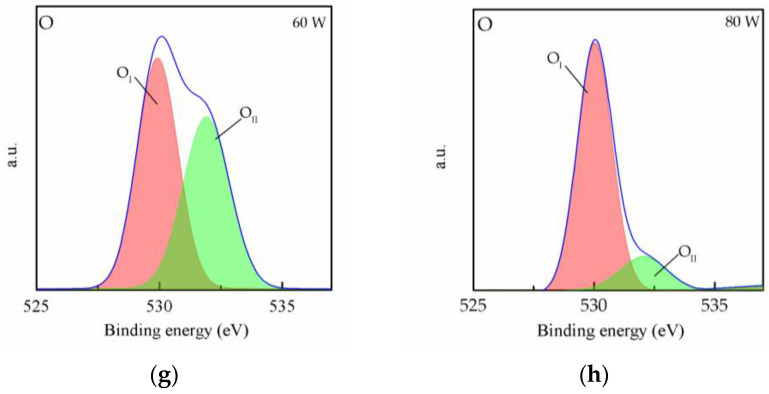
XPS spectra of MAPbI_3_ film doped with CsPbI_3_ QDs for (**a**) Pb 4f-, (**b**) I 3d- and (**c**) O 1s-treated oxygen plasma power at different powers. (**d**–**h**) Peak analyses of the XPS spectra for O 1s at different powers.

To investigate the oxygen plasma’s effect on the surface of the MAPbI_3_ film at different powers, XPS was conducted, as shown in Figure 3. Figure 3a shows the Pb 4f_7/2_ and 4f_5/2_ core level represented by two peaks centered on 137.9 and 142.8 eV, respectively. In addition, it is found that the core levels of the MAPbI_3_ film shift towards higher binding energies for 20 W curves. This result could be due to the increase in the number of oxygen ions on the surface due to the oxygen plasma treatment. Figure 3b shows the peaks of the I 3d_5/2_ and 3d_3/2_ core level. It can be observed that two peaks appear at 625 and 635 eV after oxygen plasma treatment, and the intensity of the two peaks was enhanced with the increase in the plasma powers. The results reveal the bonding of the iodine in the MAPbI_3_ film and the oxygen ions, which causes the generation of IO_3_^−^. The IO_3_^−^ improves iodine interstitial defects and results in the deep trap state being closer to the valence band [20]. Thus, the charge carrier losses can be reduced, and this means that the electric property of the MAPbI_3_ film could be enhanced by the formation of IO_3_^−^. However, the content of iodine in the film gradually decreased with increase in IO_3_^−^, as shown in Figure 3b, and this phenomenon is explained by the degradation of MAPbI_3_ due to the high plasma power. The XPS binding energy spectra for O 1s core levels are shown in Figure 3c. It can be seen that the spectra present two peaks. The O_II_ peak corresponds to the chemisorbed oxygen atoms on the surface [21], and the O_I_ peak corresponds to the bond of lead and oxygen. The O 1s XPS spectrum shows that the intensity of the O_II_ peak increased with the increase in plasma power, and it can be speculated that defects are replaced with oxygen. In order to perform further analysis, the core level spectra of O 1s were peak-fitted for each plasma power, as shown in Figure 3d–h. It can be observed that only O_II_ peak appeared for the 0 and 20 W curves, and the O_I_ peak appeared for over 40 W curves. It is conjectured that the excessive oxygen plasma power is caused the degradation of the film and the oxygen ion species bound to the lead in MAPbI_3_, as reported in other studies [22,23]. In Figure 3g,h, the intensity of the O_II_ peak is shown to drastically increase, indicating that the MAPbI_3_ was destroyed because of high power plasma. The above result is consistent with the result shown in Figure 2a.

**Figure 4:**
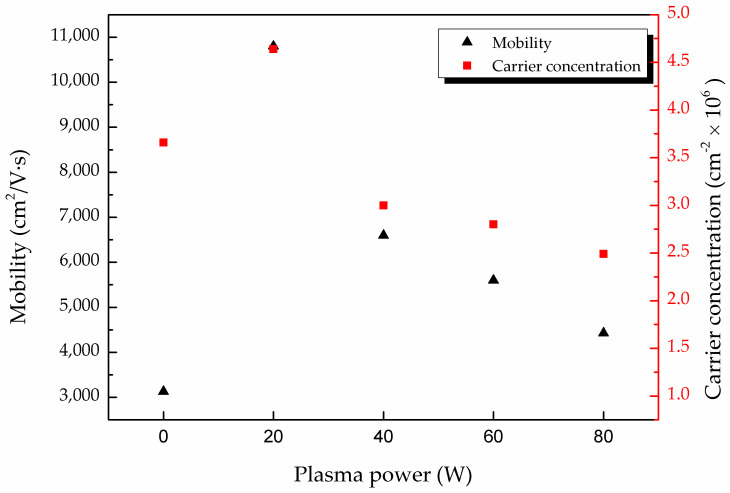
Carrier concentration (red) and carrier mobility (black) of MAPbI_3_ film doped with CsPbI_3_ QDs at various oxygen plasma powers from 0 to 80 W.

In Table 1, the mobility of the MAPbI_3_ doped with CsPbI_3_ QDs was higher than that of the pure MAPbI_3_ film. The stability could be improved by doping with CsPbI_3_ QDs, and the number of defects on the film can be decreased, leading to the higher mobility [10]. For the analysis of the conduction mechanism, the mobility and the carrier concentration were measured through Hall effect measurement. Figure 4 shows the mobility and carrier concentration of different films treated with various powers of oxygen plasma from 0 to 80 W. It can be observed that the mobility was enhanced for 20 W curves. As mentioned previously in Figure 3c, the defect was replaced by oxygen, and this leads to the improvement of mobility. Furthermore, it is shown that oxygen is a method to suppress nonradioactive recombination and to improve the photovoltaic performance [24]. The plasma treatment also caused the decrease in the grain boundary of the MAPbI_3_ film, which can be seen in a previous study [10]. With the decrease in the grain boundaries, the scattering of charge carriers reduced and the mobility of the film increased [25]. However, an excessive supply of oxygen plasma with power over 20 W caused the formation of bonds between carbon, lead and oxygen. This effect leads to the degradation of MAPbI_3_ [10,15], and therefore the mobility dropped from 6.06 × 10^3^ to 1.08 × 10^4^ cm^2^/V_s_. Compared to the untreated film, the carrier concentration of the treated MAPbI_3_ film was also enhanced. The result was attributed to the reduction in charge carrier losses, resulting from the formation of IO_3_^−^, as mentioned earlier in Figure 3b. In addition, interstitial iodine defects can decrease the carrier lifetimes of MAPbI_3_ [26], and the appearance of IO_3_^−^ reveals the passivation of interstitial iodine defects [27]. The reason for the decreased carrier concentration at higher plasma powers is the same as that for the decreased the mobility. Based on the above results, the oxygen plasma power of 20 W is the better condition.

**Figure 5:**
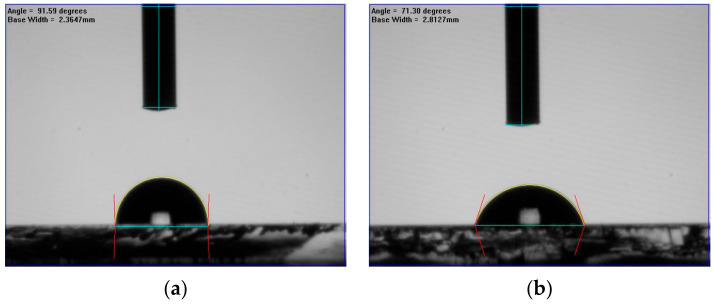

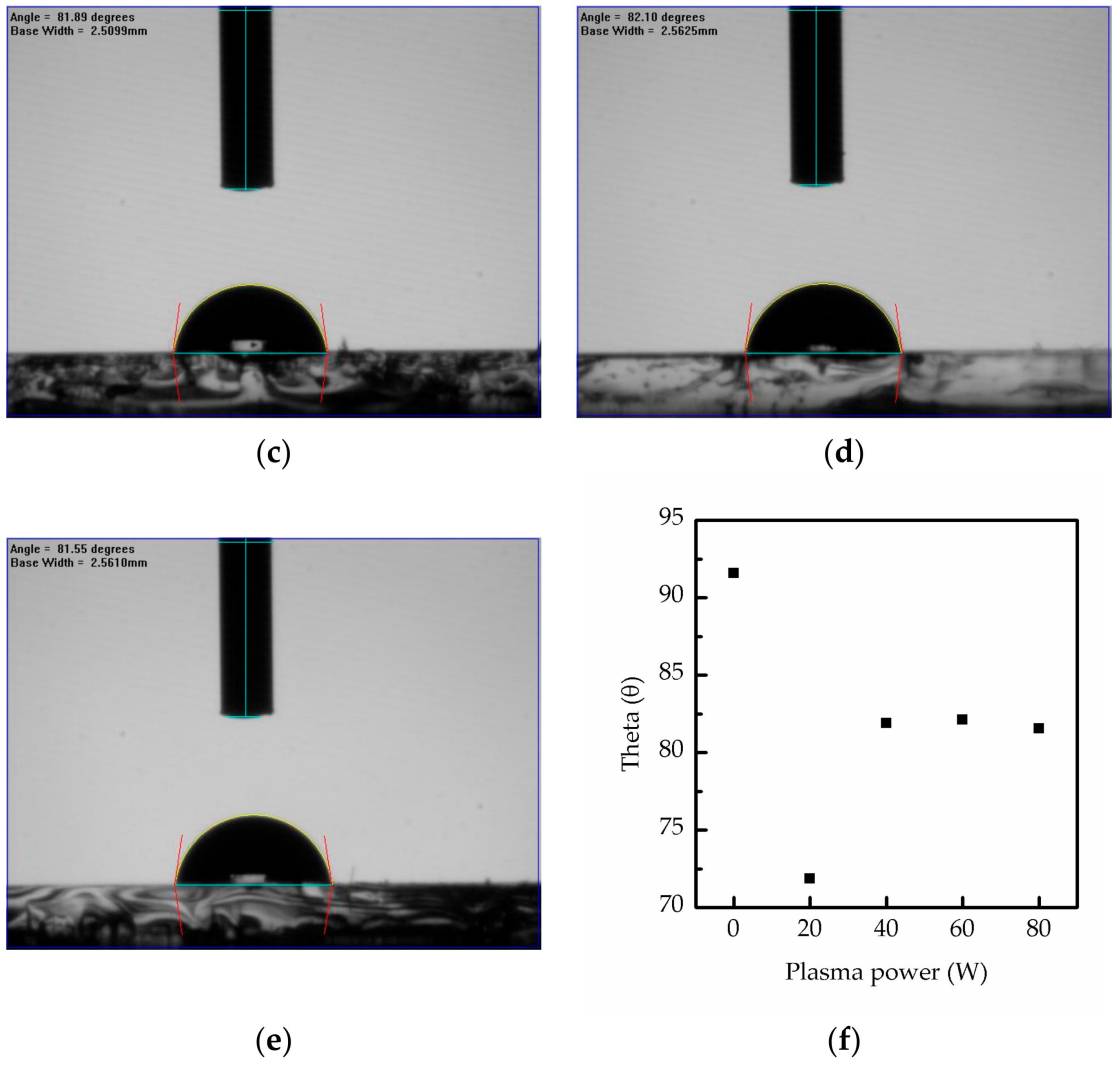
Contact angles of perovskite films composed of MAPbI_3_ and CsPbI_3_ QDs after surface treatment at various oxygen plasma powers from 0 to 80 W, (**a**) 0 W, (**b**) 20 W, (**c**) 40 W, (**d**) 60 W and (**e**) 80 W. And (**f**) the contact angles is consolidated in chart.

In Figure 5, the contact angle of the MAPbI_3_ films doped with CsPbI_3_ QDs further illustrates the effect of the oxygen plasma power and demonstrates the influence of oxygen ion bombardment. When the plasma power increased from 0 to 20 W, the contact angle of the film dropped from 91.59 degrees to 71.30 degrees. This might be due to increase in the surface energy, and the higher surface energy was caused by oxygen atoms on the surface [28]. Moreover, oxygen plasma can significantly improve surface hydrophilicity, and the increase in the hydrophilicity could be due to the carbonyl group [29,30], which might be caused by the chemisorbed oxygen atoms on the surface. However, the contact angle decreased to 81.89, 82.1 and 81.55 degrees at 40, 60 and 80 W, respectively. This means that the MAPbI_3_ is destroyed and oxygen ions started to bond with lead with the increase in plasma power. In addition, the oxygen ions also bond to the carbon in MAPbI_3_, leading to the formation of CO_x_, and the CO_x_ is not the carbonyl group on the surface, but the gas [31,32]. Although the higher plasma powers caused the degradation of the film and the formation of CO_x_, the carbonyl group still appeared on the surface. Thus, the contact angle would not change obviously at powers of 60 and 80 W.

## 3. Materials and Methods

### 3.1. CsPbI_3_ QDs Fabrication and Centrifugation

The precursor solution of QDs contained 0.4 mmol lead iodide (PbI_2_) (ACROS organic, 99%) and 0.4 mmol CsI (Alfa Aesar, Lancashire, UK, 99.9%) in oleic amine (ACROS organic, Geel, Belgium,2.4 mL) and DMF (J.T. Baker, Phillipsburg, NJ, USA, 99.5%, 10 mL). The precursor solution (0.5 mL) was quickly added into toluene (J.T. Baker, Phillipsburg, NJ, USA, 99.8%, 10 mL), while it was stirred to obtain the CsPbI_3_ QDs solution. Successively, the colloidal crude solution was centrifuged at 11,000 rpm for 15 min at 10 °C. The precipitate was collected and then successively dispersed in hexane. The above process was repeated several times.

CH_3_NH_3_I (UniRegion Bio-Tech, Hsinchu, Taiwan, 98%, 198.75 mg) and PbI_2_ (576.25 mg) were added into the mixture of 0.5 mL of sulfoxide (DMSO, J.T. Baker, 99.5%, 0.5 mg) and γ–butyrolactone (GBL, CHONEYE PURE CHEMICALS, Taipei, Taiwan, 0.5 mL, 1:1 ratio) to obtain the precursor solution. Then, this precursor solution was stirred at 300 rpm for 24 h in a glove box to obtain the perovskite MAPbI_3_ solution.

### 3.2. Fabrication of Composite Perovskite Films

CH_3_NH_3_I (50 μL) and CsPbI_3_ (1 mg) were mixed and then spin-coated onto a glass substrate in two steps, at 1000 rpm for 10 s and 5000 rpm for 20 s. Toluene was dropped on the spinning film for 15 s during the second step. Hereafter, the sample was annealed at 90 °C for 15 min to obtain the composite perovskite films. These composite perovskite films were further enhanced by oxygen plasma treatment at different powers from 0 to 80 W. The plasma treatment was carried out by radio frequency (RF) excitation with a power source of 13.56 MHz (Plasma Etch PC-150 plasma etching/cleaning system).

### 3.3. Characteristics Measurement

The absorbance spectrum of the composite perovskite films was measured by ultraviolet–visible (UV/Vis) absorption spectroscopy (U-3900 HITACHI, Tokyo, Japan). The XPS of the films was recorded using a PHI 5000 VersaProbe/Scanning ESCA Microprobe (ULVAC-PHI, Kanagawa, Japan). The top-view surface morphologies of the films were measured by field-emission scanning electron microscopy (FESEM—JEOL-6330 Cryo, Peabody, MA, USA). PL was measured by iHR350 (HORIBA, Kyoto, Japan). The TEM image was measured by JEOL-2100F CS STEM (JEOL, Tokyo, Japan). The Hall Effect was measured by L79/HCS (LENSEIS, Selb, Germany).

## 4. Conclusions

In this article, we report the effect of doping with CsPbI_3_ QDs and oxygen plasma treatment on MAPbI_3_ film. In addition, the absorbance and PL intensity was enhanced by doping with CsPbI_3_ QDs and oxygen plasma treatment. According to the Hall effect measurement, the carrier concentration and mobility was improved at 20 W. However, high powers from 40 to 80 W not only caused surface damage of the films but also destroyed the electric properties. Additionally, the influence of oxygen plasma on the surface composition was investigated by means of XPS. Moreover, the electric property was discussed by Hall effect measurement, and it is found that the mobility and carrier concertation of MAPbI_3_ was obviously enhanced through oxygen plasma treatment. According to XPS, the reason for the improvement in the mobility was also explained. In this study, we prove that oxygen plasma is a potential treatment for perovskite films.

## Figures and Tables

**Table 1:** Hall effect measurement of the pure MAPbI_3_ film and MAPbI_3_ film with CsPbI_3_ QDs treated with oxygen plasma at 0 to 80 W coated onto a glass substrate.

	Mobility (cm^2^/V_s_)	Carrier Concentration (cm^−2^)
Pure MAPbI_3_	1.95 × 10^3^	3.61 × 10^6^
0 W	3.13 × 10^3^	3.66 × 10^6^
20 W	1.08 × 10^4^	4.64 × 10^6^
40 W	6.06 × 10^3^	3.21 × 10^6^
60 W	5.60 × 10^3^	2.80 × 10^6^
80 W	4.43 × 10^3^	2.48 × 10^6^

## Data Availability

The data presented in this study are available on request from the corresponding author.

## Sample Availability

Not available.
